# Prognostic Predicting Role of Contrast-Enhanced Computed Tomography for Locally Advanced Pancreatic Adenocarcinoma

**DOI:** 10.1155/2019/1356264

**Published:** 2019-11-26

**Authors:** Chien-shan Cheng, Wei Liu, Liangping Zhou, Wei Tang, Ailing Zhong, Zhiqiang Meng, Lianyu Chen, Zhen Chen

**Affiliations:** ^1^Department of Integrative Oncology, Fudan University Shanghai Cancer Center, Shanghai 200032, China; ^2^Department of Oncology, Shanghai Medical College, Fudan University, Shanghai 200032, China; ^3^Department of Radiology, Fudan University Shanghai Cancer Center, Shanghai 200032, China; ^4^Department of Clinical Laboratory, Fudan University Shanghai Cancer Center, Shanghai 200032, China

## Abstract

**Introduction:**

Contrast-enhanced computed tomography (CECT) imaging is commonly used to assess pancreatic adenocarcinoma (PAC). However, the value of semiquantitative and quantitative assessments of CECT parameters used to predict survival in PAC remains unknown. This study aims to investigate the prognostic role of pretreatment CECT imaging in patients with locally advanced pancreatic adenocarcinoma (LAPAC).

**Materials and Methods:**

From June 2013 to May 2017, eighty-six newly diagnosed patients with pathologically and radiologically confirmed LAPAC were retrospectively recruited. All patients were evaluated by CECT and experienced gemcitabine-based chemotherapy. The relationship between overall survival (OS) and clinical factors including age, sex, serum carbohydrate antigen 19-9 value, and CECT findings (including tumour location, tumour volume, peripancreatic involvement, blood vessel involvement, tumour enhanced rate, and distance liver metastasis) was determined using Cox proportional hazard regression models, and a nomogram was constructed for the prediction of 1- and 1.5-year survival rates of patients with LAPAC.

**Results:**

On univariate analysis, patients who had a tumour enhanced rate (TER) less than 80.465% and those who had a TER ≥ 80.465% are with a 3.587-fold increase in OS (*p* < 0.001). After multivariate Cox regression, a nomogram was established based on a new model containing the predictive variables of high Ca19-9 level, higher clinical stages, larger tumour volume, the presence of peripancreatic involvement, and liver metastases. The model displayed good accuracy in predicting OS with a C-index of 0.614. The calibration plots also showed a good discrimination and calibration of the nomogram between the predicted and observed survival probabilities.

**Conclusion:**

Our results showed that TER can be used to predict survival in LAPAC, and we developed a nomogram for determining the prognosis of patients with LAPAC. However, the purposed nomogram still requires external data verification in future applications.

## 1. Introduction

Pancreatic adenocarcinoma (PAC) is one of the most lethal malignancies and is estimated to cause more than 45,750 deaths in the United States in 2019 [1]. Due to the lack of particularly high-risk factors or early detection tests, more than 40% of patients are diagnosed with locoregionally advanced (stage III/IV) disease when present, resulting in a median survival of 6 to 11 months [2, 3]. As the survival rates vary considerably among patients with LAPAC, there is an urgent need to identify new markers that can help predict patient survival to tailor future treatments and predict the prognosis for LAPAC patients [4].

For malignant tumour, traditional prognostic factors are mainly clinical-pathological features such as performance status, histological grade, serum biomarkers, systemic inflammation and nutritional status, and TNM staging, which are frequently evaluated by contrast-enhanced computed tomography (CECT) and/or magnetic resonance (MR) imaging [5, 6]. In PAC, CECT and its imaging reconstruction are widely used in the diagnosis, treatment planning, and preoperative assessment of surgical resectability [7]. Tumour hypoxia and inadequate vascularization are well-established biological phenomena that are linked to poor patient outcomes. Overexpression of hypoxia-inducible factor 1*α* (HIF-1*α*), an endogenous marker of hypoxia, is significantly associated with poor prognosis in PAC [8]. However, most LAPAC patients have unresectable disease and have not undergone surgical resection, making it difficult to obtain histological specimen to assess HIF-1*α* expression. Although a small amount of pathological tissue can be obtained by biopsy, the expression of HIF-1*α* cannot accurately reflect the hypoxic state of the tumour due to the heterogeneity of the tumour. Studies have shown that HIF-1*α* is negatively correlated with microvessel density (MVD) [9, 10]. In addition, the degree of CECT contrast enhancement is related to the MVD count in the PAC and the degree of tumour malignancy [9]. Therefore, we hypothesize that the degree of enhancement achieved by CECT may reflect the prognosis of advanced pancreatic cancer to some extent. In accordance with the Reporting Recommendations for Tumour Marker Prognostic Studies (REMARK) [11], this present study aims to evaluate the utility of CECT in predicting prognosis in LAPAC patients, to explore whether CECT parameters can synergistically predict prognosis with clinical indicators through the Cox proportional hazard regression models and the nomogram model.

## 2. Materials and Methods

### 2.1. Patients

**Figure 1:**
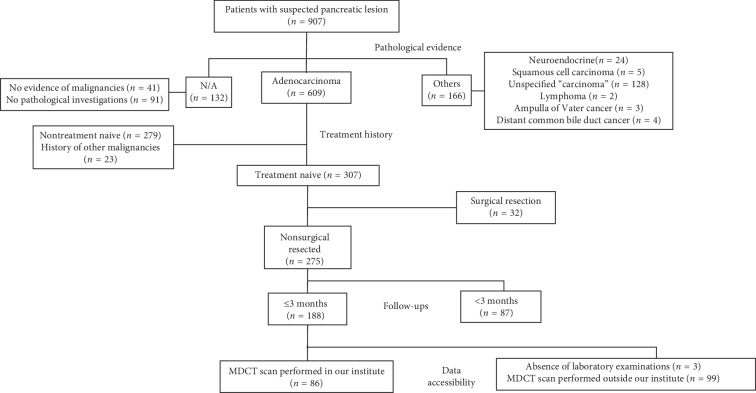
Patient inclusion flow. Clinical data collected from medical charts and follow-up records include age, gender, height, weight, body mass index, tumour histology, baseline laboratory values (complete blood count, hepatic and renal function, and serum chemistry), serum tumour marker carbohydrate antigen 19-9 (Ca19-9), and survival status. This study was carried out in accordance with the Helsinki Declaration, and approvals were obtained from the ethics committee of our medical institution. Written informed consent was obtained from every included patient or their guardians for the use of clinical data.

We retrospectively reviewed the medical records of 907 patients with pancreatic lesion admitted in our institute from 1 June 2013 to 31 May 2017. Among this population, the selection criteria for data extraction were as follows: (1) patients who had pathologically proven pancreatic adenocarcinoma; (2) adequate quality of the abdominal contrast-enhanced computed tomography (CT) scan; (3) no history of malignancies of other origin; (4) the availability of laboratory examinations; (5) follow-up for more than three months after the initial diagnosis. The exclusion criteria included patients with neuroendocrine carcinoma of the pancreas, ampulla Vater cancer, or distal common bile duct cancer and patients with a previous history of treatment for PAC. We identified a total of 188 patients and extracted related medical records and data. We further excluded 102 patients among the identified patients due to either the absence of pretreatment CECT scans performed in our institute or absence of multiphase contrast scan. Finally, 86 patients with LAPAC, who all experienced gemcitabine-based chemotherapy, were screened out for analysis. Inclusion details are shown in Figure 1.

### 2.2. Imaging Analysis

All CECT images were obtained with multidetector CT scanners (Sensation 64, Siemens Medical System), obtained at 120 kV and 200 mA with collimation of 1 mm and 3 mm reconstruction thickness. Plain and multiphase contrast images were obtained following general clinical settings. Contrast images were acquired in the arterial phase and the venous phase. The delay time for the contrast-enhanced CT images was 35–40 seconds for the arterial phase and 65–70 seconds for the venous phase after the intravenous injection of 80–100 mL Iohexol (Omnipaque 300; General Electric) at a rate of 3 mL/s. CECT parameters including tumour location, tumour volume (TV), peripancreatic involvement (PPI), blood vessel involvement (BVI), tumour enhanced rate (TER), peritumoural pancreatic parenchyma enhanced rate (PER), and the presence of liver metastasis were evaluated by two radiologists independently. The TV was calculated based on an imaging reconstruction tool (GE AW SERVER 2.0). The contrast-enhanced CT value of tumour and nontumoural pancreatic tissue was measured at the venous phase. The region of interest (ROI) was defined in the solid portion of the mass and pancreatic parenchyma and bypassed peripheral fat, artefacts, necrosis, cystic necrosis/cystic components, and blood vessels and pancreatic ducts. ROI with an area of approximately 50 mm^2^ was delineated, respectively, in three different solid slices for each tumour and peritumoural pancreatic tissue, and then, three CT values were achieved to compute the mean CT value. TER was computed as equation (1). PER was computed as equation (2):

(1) $$\begin{array}{c} \text{TER}=\frac{\text{venous phase enhanced CT value of tumour}-\text{plain CT value of tumour}}{\text{plain CT value of tumour}}, \end{array}$$

(2) $$\begin{array}{c} \text{PER}=\frac{\text{venous phase enhanced CT value of pancreas}-\text{plain CT value of pancreas}}{\text{plain CT value of pancreas}}. \end{array}$$

**Figure 2:**
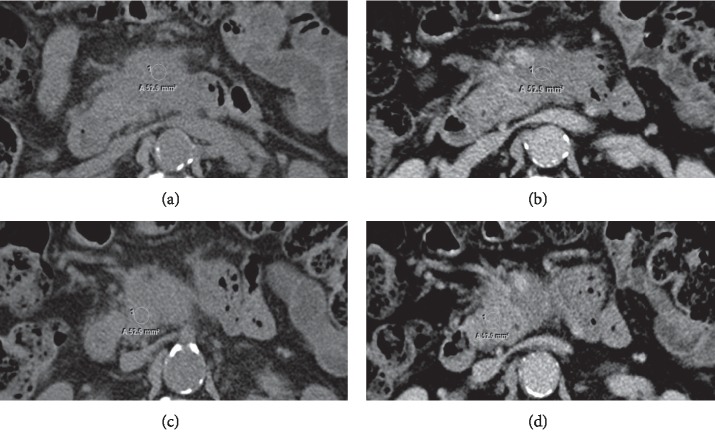
Illustration of the obtained methods of measuring the region of interest (ROI). A 69-year-old male patient with pancreatic adenocarcinoma in uncinate process of pancreas. (a) ROI of tumour in plain CT. (b) ROI of tumour in venous phase enhancement CT. (c) ROI of peritumour in plain CT. (d) ROI of peritumour in venous phase enhancement CT.

**Figure 3:**
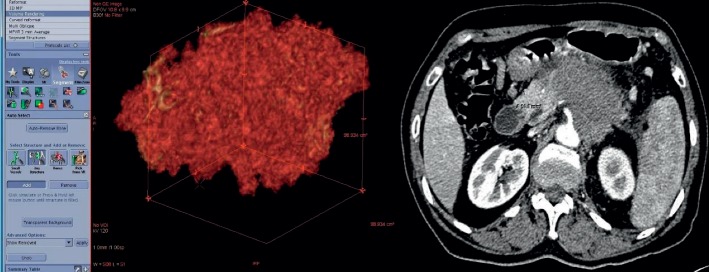
Measure of tumour volume (TV) by the imaging reconstruction tool. An illustration of a 65-year-old male patient with pancreatic adenocarcinoma in the pancreatic body.

Figures 2 and 3 illustrate the acquisition methods of ROI and TV, respectively.

### 2.3. Statistical Analysis

Continuous variables were analysed nonparametrically using the Mann–Whitney *U* test. The categorical variables were analysed by the chi-squared test or Student's *t*-test, where appropriate. Based on the 75^th^ percentile of TER, patients with LAPAC were divided into a relatively abundant blood supply group (TER ≥ 80.465%) and a relatively poor blood supply group (TER < 80.465%). A receiver operating characteristic (ROC) curve was calculated to determine the optimal cutoff value for the TV based on the six-month survival rate. Univariate prognostic factor analysis was performed using the Cox proportional hazard model to estimate the hazard ratio (HR) and 95% confidence interval (CI). Factors associated with survival in the univariate analysis entered into a final multivariate survival analysis with a stepwise Cox regression model for identifying independent prognostic factors. Based on multivariate analyses, nomograms were generated to provide visualized risk prediction using the survival. Discrimination between survival probability and actual observations was evaluated using the C-index. Statistical analyses were performed using SPSS 21.0 (SPSS, Chicago, IL, USA) or the package of *rms* in R software version 2.15.2 (R Development Core Team; http://www.r-project.org). Values of *p* less than 0.05 were considered statistically significant, and all tests were two-sided.

## 3. Results

### 3.1. Patient Characteristics

Patient baseline characteristics and CT imaging parameters of the 86 treatment naïve patients are shown in Table 1. The mean patient age was 60.17 ± 7.82 years. The mean patient body mass index (BMI) was 22.83 ± 2.70. The clinical stages classifications for IIb, III, and IV were 18 (20.9%), 27 (31.4%), and 41 (47.7%) patients, respectively. The median TV was 26,411 mm^3^ (range, 3401–125,187 mm^3^). Peripancreatic invasion (PPI) was identified in 6 patients (7.0%). The median TER was 53.21% (range, 17.00%–169.980%). The median PER was 132.30% (range, 53.00%–194.40%). The median overall survival was 6.05 months (range, 1.40–21.40 months).

### 3.2. Correlation of Tumour Enhanced Rate with Clinical Stages

We further evaluated the correlation between TER and the clinical stages of pancreatic cancer. TER is different among clinical stages, and Pearson correlation analysis suggested that clinical stages were negatively correlated with TER (*r* = −0.470, *p* < 0.001). The chi-squared test showed a statistically significant difference in staging of patients with different TERs (*p* < 0.001). The more advanced tumour clinical stage was mainly distributed in the TER < 80.465 group. These results suggest that TER is associated with the clinical stages in pancreatic cancer.

### 3.3. Risk Factor for Poor Prognosis

Data on sex, age, BMI, a family history of malignancies, tumour location, clinical stages, serum Ca19-9, the presence of pancreatic duct dilation (PDD), bile duct dilation (BDD), liver metastasis, TV, and TER were collected. Univariate analysis indicates that more advanced clinical stages (*p* < 0.001), presence of PPI (*p* < 0.05), presence of liver metastases (*p* < 0.001), TER lower than 80.465% (*p* < 0.001), TV higher than 24,062.25 (mm^3^) (*p* < 0.001), and Ca19-9 ≥1000 (IU/mL) (*p* < 0.001) significantly predict a poor prognosis, as shown in Table 2. Multivariable analysis indicates that more advanced clinical stages and Ca19-9 ≥1000 (IU/mL) are significant factors for poor prognosis (*p* value <0.001 and <0.005, respectively), as shown in Table 3, while TER ≥ 80.465% (*p* < 0.001) is a significant factor for favourable prognoses. TER ≥ 80.465% group enjoys survival benefit (median OS = 13.80 ± 5.71 months) over TER < 80.465% group (median OS = 5.60 ± 3.36 months) (*p* < 0.001). TER significantly predicts OS in both univariate and multivariate analyses (*p* < 0.001). The predicting model for poor prognosis in patients with LAPAC is as follows:

(3) $$\begin{array}{c} Y=\frac{h\left(t\right)}{h\left(t_{0}\right)}=\exp\left(2.42*\text{Ca}19-9+\;2.375*\text{ clinical stages }–\;3.587*\text{TER}\right). \end{array}$$

### 3.4. Development of Prognostic Nomogram for Overall Survival and the Evaluation of the Calibration Curve

**Figure 4:**
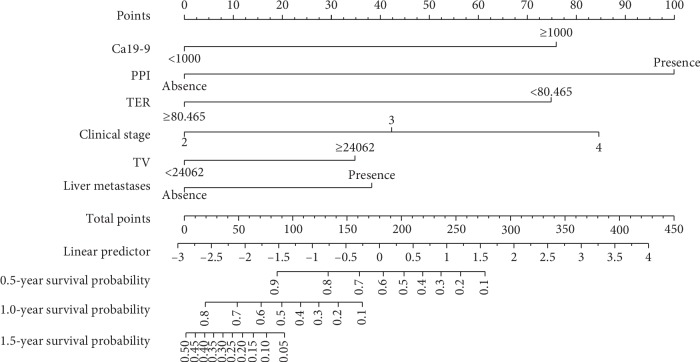
Nomogram of multifactor Cox regression survival probability in patients with locally advanced pancreatic adenocarcinoma. Nomograms for predicting survival probability based on the derivation set. This nomogram is used by adding up the score of each individual variable identified on the scale. According to the total points projected on the bottom scales, the nomogram can provide the probabilities of 0.5-, 1.0-, and 1.5-year of survival an individual patient. PPI, peripancreatic involvement; TER, tumour enhanced rate; TV, tumour volume.

**Figure 5:**
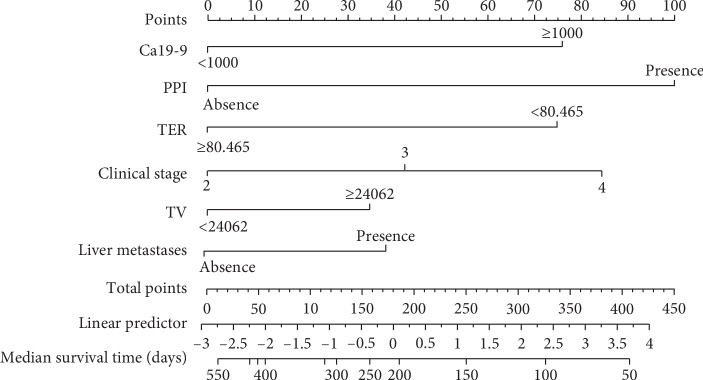
Nomogram predicting factors for median survival in patients with locally advanced pancreatic adenocarcinoma. Nomograms for predicting the median survival time based on the derivation set. This nomogram is used by adding up the score of each individual variable identified on the scale. According to the total points projected on the bottom scales, the nomogram can provide the probabilities of median survival time in days of an individual patient. PPI, peripancreatic involvement; TER, tumour enhanced rate; TV, tumour volume.

**Figure 6:**
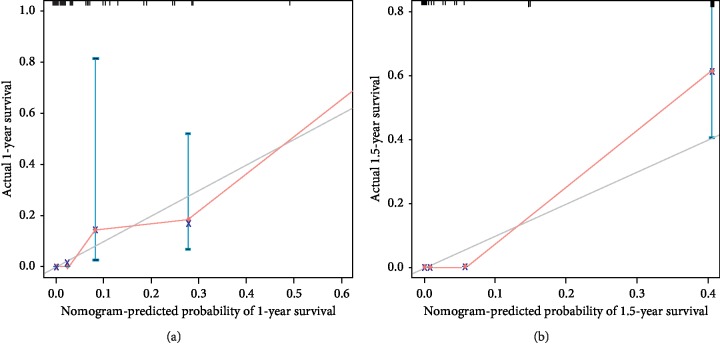
Calibration curves for predicting patient OS. Calibration curves for predicting 1-year survival (a) and 1.5-year survival (b). The *Y*-axis demonstrated the actual while the *X*-axis demonstrated the nomogram-predicted survival probabilities. A closer distance between two curves in red indicates higher accuracy of prediction.

The univariate and multivariate Cox regression models with a stepwise selection procedure identified the following variables: Ca19-9, the presence of PPI, TER, tumour volume, and the presence of liver metastasis. The individual variable was assigned a score, and the total score for all variables on the total point scale, the 0.5-year, 1.0-year, and 1.5-year survival probabilities, as well as median survival time, was determined by drawing a vertical line from the total score (Figures 4 and 5). The calibration curve and C-index were used to assess the discrimination ability of the nomogram, and the C-index for the overall survival prediction was 0.613 suggesting a good agreement between the nomogram predictions and actual 1.0- and 1.5-year survival rates (Figure 6).

## 4. Discussion

PAC is the fourth leading cause of cancer-related death and has an overall 5-year survival rate of <5% due to the high risk of recurrence, metastasis, and the relative chemoresistance [3]. Surgical resection is the only curative treatment option, but >80% of patients are diagnosed at inoperable clinical stages. Identifying patients at high risk of poor survival would play an important role in making projects of palliative therapy. For patients predicted with a poor prognosis, providing more palliative care may provide survival benefits, and for those predicted with better prognosis, appropriately reduced palliative treatment may be considered to restrain adverse reactions. In the present study, we developed a semiquantitative assessment of CECT parameters combined with some clinical features to predict survival in patients with LAPAC. We found that TER, the presence of PPI, TV, the presence of liver metastasis, and elevated Ca19-9 are independent prognostic factors for OS in patients with LAPAC.

In our study, a low TER was significantly associated with short survival, which is consistent with the study of Wang et al. [9]. Another previous study also assessed PAC by contrast-enhanced ultrasonography and found that low tumour vascularity predicts poor prognosis in treatment naïve LAPAC patients (*p* < 0.001) [12]. Low MVD and the overexpression of the vascular endothelial growth factors (VEGF) are associated with hypoxia, which can predict a poor prognosis of PAC [8]. In addition, high blood perfusion of PAC may be correlated with the well differentiation of tumours [13, 14]. Wang et al. also found that the degree of CT enhancement was negatively correlated with the pathological grade of PAC [9]. Furthermore, the initial treatment of patients in our study was gemcitabine-based chemotherapy. High TER indicates a relatively higher vascularity and abundant tumour blood supply, which may facilitate drug delivery and distribution to the tumour tissue, resulting in the better therapeutic outcomes of gemcitabine-based chemotherapy. The above findings can be approached with our cautious results.

Recent studies have also reported that hypoxic microenvironment plays a critical role in the acquired chemoresistance of pancreatic cancer cells [15, 16]. Under the hypoxic condition, PAC cells are more resistant to gemcitabine-induced apoptosis than under the normoxic condition [15], and silencing HIF-1*α* of the human pancreatic cancer cell can reverse the chemotherapy drug resistance [16]. Moreover, gemcitabine promoted pancreatic cancer cell stemness, and the ubiquitous hypoxic niche accounted for drug resistance and associated with malignant phenotypes such as enhanced migration, invasion, and metastasis [17]. Therefore, it is possible that low TER, which may reflect low microvascular supply and a more hypoxic tumour microenvironment, may result in chemoresistance and a poor prognosis in patients with locally advanced pancreatic carcinoma undergoing systemic chemotherapy. However, it is unclear of concrete mechanism that low vascularity in tumours is associated with chemoresistance and poor prognosis. Further experimental and clinical studies are needed to conclude the significance of pretreatment tumour vascularity in patients with pancreatic cancer.

In contrast, another study showed tumour vascularity assessed by contrast-enhanced ultrasonography before treatment did not correlate with progression-free survival nor with overall survival [18]. The discrepancy may be due to the method of assessment of whether the whole or partial tumour was examined, as some studies have shown that the difference among intratumour microvessel and peripheral microvessel density may result in the vascular heterogeneity of pancreatic cancer [19]. Further, the patients' demography may also contribute to the variance among the assessment of the correlation between the tumour vascularity and the prognosis.

In our study, univariate Cox regression analysis found that the presence of PPI, Ca19-9, TER, TV, clinical stages, and the presence of liver metastasis are statistically significant prognostic factors. However, TV and the presence of liver metastasis did not retain prognostic roles in further multivariate Cox regression. These may result from our interpretation that clinical staging is based on TNM staging, which is a combination of tumour size, the presence of local invasion, local lymph node status, and distant metastasis. There is a strong correlation between TV and liver metastasis and clinical stages. Therefore, TV and the presence of liver metastasis are not independent prognostic factors. This may provide an explanation to the difference in statistically significant prognostic factors between univariate and multivariate analyses.

A nomogram can integrate several risk factors and provides a more individualized prediction of prognosis and risk assessment for each patient. Compared with the conventional staging system, a nomogram model increased prediction accuracy in some neoplasms such as urothelial carcinoma, intrahepatic cholangiocarcinoma, and breast cancer [20–22]. Our final nomogram integrated five parameters, including serum Ca19-9, the presence of PPI, TER, tumour volume, and the presence of liver metastasis, while among them, four of the five parameters can be obtained from CECT findings. To some degree, our results are similar to previous studies [23–25]. Our model shows a high consistency with daily clinical practice where serum Ca19-9 is used for screening the disease and CECT examination for the identification of the resectability of pancreatic cancer. Moreover, our nomogram demonstrated a high discriminatory ability with a C-index of 0.763 with the actual observation. In subsequent studies, it will be of value to assess the clinical utility and the prediction precision of the nomogram.

However, in the present study, as with most retrospective studies, our study has a limitation. Further, the absolute number of total subjects studied is relatively small in this study, and the nomogram was constructed without a division for a validation cohort. We, therefore, analysed the C-index of the constructed nomogram and the calibration curve fit at the different time periods of prediction well between nomogram prediction and the actual observation. Moreover, our measurement of TER reflected a relative change of CT value in plain CT and enhanced venous phase instead of an absolute perfusion parameter. This problem can be solved by drawing a time-contrast dose curve by repeated sequential scans of each tumour slice followed by calculating the area under the curve. However, further studies on providing an absolute tumour perfusion parameter may be limited as obtaining this parameter will require a high radiation dose by scanning each one of the CT slices. Dynamic contrasted enhancement MR will address this problem and can provide more perfusion parameters so that further research studies can begin with this respect. Thus, the findings of this study must be interpreted with caution.

## 5. Conclusions

Taken together, our results indicate that tumour enhanced rate on CECT demonstrated prognostic significance in univariate analysis and maintained its significant roles in multivariable analysis. Our results suggest that pretreatment CECT scans can be used to predict survival in advance pancreatic cancer.

## Figures and Tables

**Table 1:** Clinical characteristics and CECT parameters.

Characteristic	Total (*n* = 86)	High TER ≥80.465 *n* = 21 (24.4%)	Low TER <80.465 *n* = 65 (75.6%)	*P*
Age (years)				
Mean (SD)	60.17 (7.82)	63.67 (7.49)	59.05 (7.64)	0.017

Gender, *n* (%)				
Men	59 (68.6%)	13	46	0.447
Women	27 (31.4%)	8	19	

BMI (kg/m^2^)				
Mean (SD)	22.83 (2.70)	22.35 (3.34)	22.99 (2.47)	0348

Family history, *n* (%)	18 (20.9%)	2	16	0.139
Clinical stages, *n* (%)				
IIb	18 (20.9%)	12	6	**<0.001**
III	27 (31.4%)	5	22	
IV	41 (47.7%)	4	37	

Tumour location, *n* (%)				
Head and neck	32 (37.2%)	9	23	0.538
Body and tail	54 (62.8%)	12	42	

Ca19-9 (IU/mL)				
Mean (SD)	665.3 (415.2)	659.4 (373.7)	667.2 (430.5)	0.959
<1000, *n* (%)	40 (46.5)	15	25	
≥1000, *n* (%)	46 (53.5)	17	29	

Overall survival (months)				
Range	1.40–21.40	3.80–21.40	1.40–18.86	
Median (SD)	6.05 (5.45)	13.80 (5.71)	5.60 (3.36)	**<0.001**

Liver metastases, *n* (%)				
Presence	38 (44.19%)	2	36	**<0.001**
Absence	48 (55.81%)	19	29	

Tumour volume (mm^3^)				
Range	3401–125187	3401–23186	3750–125187	**0.011**
Mean (SD)	26411 (26386)	13848 (6633)	30469 (29024)	

PPI, *n* (%)	6 (7.0%)	0	6	0.149

BVI, *n* (%)	50 (58.1%)	13	37	0.687

PDD, *n* (%)	36 (41.9%)	10	26	0.538

BDD, *n* (%)	10 (11.6%)	2	8	0.729

PER (%)				
Range	53.00–194.40	73.80–194.40	53.00–181.62	0.071
Median (SD)	132.30 (37.86)	157.39 (41.36)	123.51 (35.85)	

TER (%)				
Range	17.00–169.80	81.21–169.80	17.00–79.72	
Median (SD)	53.21 (38.07)	105.76 (28.95)	42.34 (21.06)	

Bolded text indicates a statistically significant difference with a *p* value less than 0.05. BMI, body mass index; family history, a family history of malignancies; Ca19-9, carbohydrate antigen 19-9; PPI, peripancreatic involvement; BVI, blood vessel involvement; PDD, pancreatic duct dilation; BDD, bile duct dilation; PER, peritumoural pancreatic parenchyma enhanced rate; TER, tumour enhanced rate; SD, standard deviation.

**Table 2:** Univariate analysis of predicting factors for overall survival in patients with locally advanced pancreatic cancer.

Variables	B	SE	Wald	Sig.	Exp(B)	95.0% CI for exp(B)	
						Lower	Upper
Male gender	−0.187	0.306	0.374	0.541	0.829	0.455	1.511
Age ≥60 (years)	0.151	0.285	0.282	0.595	1.164	0.665	2.035
BMI <22.8 (kg/m^2^)	0.734	0.262	7.815	0.005	2.083	1.245	3.484
Family history of malignancies	0.072	0.348	0.042	0.837	1.074	0.543	2.126
Presence of PDD	0.34	0.308	1.215	0.27	1.405	0.768	2.57
Presence BDD	−0.072	0.481	0.022	0.881	0.93	0.362	2.39
Presence of BVI	−0.207	0.305	0.461	0.497	0.813	0.447	1.478
Tumour located at head and neck	−0.836	0.379	4.86	0.027	0.434	0.206	0.911
Presence of PPI	−0.004	0.663	0	**0.012**	0.996	0.271	3.655
Ca19-9 ≥1000 (IU/mL)	1.294	0.343	14.248	**<0.001**	3.649	1.863	7.145
TER ≥80.465 (%)	−1.706	0.452	14.256	**<0.001**	0.182	0.075	0.44
Tumour volume	0.531	0.395	1.812	**<0.001**	1.701	0.785	3.685
Clinical stages	0.485	0.321	2.292	**<0.001**	1.625	0.867	3.045
Presence of liver metastasis	1.209	0.474	6.517	**<0.001**	3.351	1.324	8.478

Bolded text indicates a statistically significant difference with a *p* value less than 0.05. BMI, body mass index; PPI, peripancreatic involvement; BVI, blood vessel involvement; PDD, pancreatic duct dilation; BDD, bile duct dilation; TER, tumour enhanced rate; Ca19-9, carbohydrate antigen 19-9; CI, confidence interval.

**Table 3:** Multivariate analysis of predicting factors for overall survival in patients.

Variables	B	SE	Wald	Sig.	Exp(B)	95.0% CI for exp(B)	
						Lower	Upper
TER ≥80.465 (%)	−1.72	0.377	20.773	**<0.001**	0.179	0.085	0.375
Ca19-9 ≥1000 (IU/mL)	0.884	0.284	9.67	**0.002**	2.42	1.386	4.224
Clinical stages	0.865	0.19	20.647	**<0.001**	2.375	1.636	3.45
Presence of PPI	0.791	0.528	2.242	0.134	2.206	0.783	6.216
Tumour volume	0.412	0.302	1.856	0.173	1.51	0.835	2.731
Presence of liver metastasis	0.452	0.402	1.264	0.261	1.572	0.714	3.459

Bolded text indicates a statistically significant difference with a *p* value less than 0.05. TER, tumour enhanced rate; Ca19-9, carbohydrate antigen 19-9; PPI, peripancreatic invasion; CI: confidence interval.

## Data Availability

The data used to support the findings of this study are available from the corresponding author upon request.

## References

[1] Siegel R. L., Miller K. D., Jemal A. (2019). Cancer statistics, 2019. *CA: A Cancer Journal for Clinicians*.

[2] Pietryga J. A., Morgan D. E. (2015). Imaging preoperatively for pancreatic adenocarcinoma. *Journal of Gastrointestinal Oncology*.

[3] Siegel R. L., Miller K. D., Jemal A. (2017). Cancer statistics, 2017. *CA: A Cancer Journal for Clinicians*.

[4] Wang P., Zhuang L., Zhang J. (2013). The serum miR-21 level serves as a predictor for the chemosensitivity of advanced pancreatic cancer, and miR-21 expression confers chemoresistance by targeting FasL. *Molecular Oncology*.

[5] Bilici A. (2014). Prognostic factors related with survival in patients with pancreatic adenocarcinoma. *World Journal of Gastroenterology*.

[6] Wang P., Fan J., Chen Z. (2009). Low-level expression of Smad7 correlates with lymph node metastasis and poor prognosis in patients with pancreatic cancer. *Annals of Surgical Oncology*.

[7] Karmazanovsky G., Fedorov V., Kubyshkin V., Kotchatkov A. (2005). Pancreatic head cancer: accuracy of CT in determination of resectability. *Abdominal Imaging*.

[8] Ye L.-Y., Zhang Q., Bai X.-L., Pankaj P., Hu Q.-D., Liang T.-B. (2014). Hypoxia-inducible factor 1*α* expression and its clinical significance in pancreatic cancer: a meta-analysis. *Pancreatology*.

[9] Wang S. H., Sun Y. F., Liu Y., Zhou Y., Liu Y. (2015). CT contrast enhancement correlates with pathological grade and microvessel density of pancreatic cancer tissues. *International Journal of Clinical and Experimental Pathology*.

[10] Couvelard A., O’Toole D., Turley H. (2005). Microvascular density and hypoxia-inducible factor pathway in pancreatic endocrine tumours: negative correlation of microvascular density and VEGF expression with tumour progression. *British Journal of Cancer*.

[11] Sauerbrei W., Taube S. E., McShane L. M., Cavenagh M. M., Altman D. G. (2018). Reporting recommendations for tumor marker prognostic studies (REMARK): an abridged explanation and elaboration. *JNCI: Journal of the National Cancer Institute*.

[12] Masaki T., Ohkawa S., Amano A., Ueno M., Miyakawa K., Tarao K. (2005). Noninvasive assessment of tumor vascularity by contrast-enhanced ultrasonography and the prognosis of patients with nonresectable pancreatic carcinoma. *Cancer*.

[13] Suga H., Okabe Y., Tsuruta O. (2014). Contrast-enhanced ultrasonograpic studies on pancreatic carcinoma with special reference to staining and muscular arterial vessels. *The Kurume Medical Journal*.

[14] Nanashima A., Shibata K., Nakayama T. (2012). Relationship between microvessel count and clinicopathological characteristics and postoperative survival in patients with pancreatic carcinoma. *Hepatogastroenterology*.

[15] Yokoi K., Fidler I. J. (2004). Hypoxia increases resistance of human pancreatic cancer cells to apoptosis induced by gemcitabine. *Clinical Cancer Research*.

[16] Yang S. Y., Song B. Q., Dai S. L., Yang K. X., Jin Z., Shi K. W. (2014). Effects of hypoxia-inducible factor-1alpha silencing on drug resistance of human pancreatic cancer cell line Patu8988/5-Fu. *Hepatogastroenterology*.

[17] Zhang Z., Han H., Rong Y. (2018). Hypoxia potentiates gemcitabine-induced stemness in pancreatic cancer cells through AKT/notch1 signaling. *Journal of Experimental & Clinical Cancer Research*.

[18] Tawada K., Yamaguchi T., Kobayashi A. (2009). Changes in tumor vascularity depicted by contrast-enhanced ultrasonography as a predictor of chemotherapeutic effect in patients with unresectable pancreatic cancer. *Pancreas*.

[19] Takagi K., Takada T., Amano H. (2005). A high peripheral microvessel density count correlates with a poor prognosis in pancreatic cancer. *Journal of Gastroenterology*.

[20] Necchi A., Sonpavde G., Lo Vullo S. (2017). Nomogram-based prediction of overall survival in patients with metastatic urothelial carcinoma receiving first-line platinum-based chemotherapy: retrospective international study of invasive/advanced cancer of the urothelium (RISC). *European Urology*.

[21] Orucevic A., Bell J. L., McNabb A. P., Heidel R. E. (2017). Oncotype DX breast cancer recurrence score can be predicted with a novel nomogram using clinicopathologic data. *Breast Cancer Research and Treatment*.

[22] Wang Y., Li J., Xia Y. (2013). Prognostic nomogram for intrahepatic cholangiocarcinoma after partial hepatectomy. *Journal of Clinical Oncology*.

[23] Vennin C., Murphy K. J., Morton J. P., Cox T. R., Pajic M., Timpson P. (2018). Reshaping the tumor stroma for treatment of pancreatic cancer. *Gastroenterology*.

[24] Ruiz-Tovar J., Martin-Perez E., Fernandez-Contreras M. E., Reguero-Callejas M. E., Gamallo-Amat C. (2011). Identification of prognostic factors in pancreatic cancer. *Cirugía y Cirujanos*.

[25] Zhang D.-X., Dai Y.-D., Yuan S.-X., Tao L. (2012). Prognostic factors in patients with pancreatic cancer. *Experimental and Therapeutic Medicine*.

